# Stammering Considered with Reference to Its Cure

**Published:** 1836-10-01

**Authors:** 


### Stammering considered with reference to its Cure, by the Application of those Laws which regulate Utterance. In a Letter addressed to George Birkbeck, M.D. &c. &c. &c. By *Richard Cull.* Octavo, pp. 50, stitched. London, 1835.

It would be tedious to enumerate the various modes that ignorance, empiricism, and imposture have at various times proposed for the cure of impediments of speech. From the farmer in Joe Miller, who made his son sing—"Daddy your house is on fire," down to Mrs. Leigh, the Lady in New York, who forces her disciples to keep the tongue against the teeth uninterruptedly for three days, the plans have been innumerable, the proposers confident, and the success, of course, immense. Yet, singularly as it would seem, although each plan is perfectly successful, the one that follows is advanced expressly, because no such thing as success can be obtained. How this paradoxical state of things may be explained, we leave to others to determine.

The object of the author of the present pamphlet is not to set forth the infallibility of any one specific—of taking a deep or a short inspiration—of keeping the tongue up or keeping it down—of squaring all sounds by one vowel or one consonantic sound—of talking incessantly or not talking at all, but his aim is wider, his views more philosophical, and both may broadly be said to be—the education of the voice. We shall not enter into Mr. Cull's details, partly because they would occupy too much time, and partly because the majority of our readers would perhaps be hardly able to understand the somewhat learned terms he uses. In describing, for instance, the voice of a stammerer, he observes:—

> "The portions of voice are about minims in duration—staccato on a rapid diminuendo of a wide interval downward in flexion, so that a word of only two elements is imperfectly formed." 42.

These are hard words, and abstruse to the uninitiated. Indeed were we disposed to hint a fault, we should say that Mr. Cull's practices in the elocutionary way had given his style of writing somewhat of King Cambyses' vein, or a little of the swagger of Ancient Pistol. But such faults of manner are slight blemishes only, and the character of the observations is characterized on the whole by good sense, and a philosophical spirit. We shall endeavour to give a slight sketch of the views and the advice of Mr. Cull.

He shews that stammering may depend on various causes; in other words, that as many parts are concerned in the production of vocal sounds and of speech, and as many individual actions are necessary in order to render it uninterrupted and perfect, the derangement of any, or the want of consent between all, may lead to the production of stammering.

> "There are about twenty elements of speech productive of an interruption in various degrees of the continuity of the voice, and by their mingling with the remainder in our language, one occurs at about every fifth place. Although a person may stammer on all the twenty, yet from what has just been said, he does not necessarily hesitate at every fifth element, as the interruption depends on several other circumstances—as the mechanism of the element enunciated, and the element on which the articulation takes place; and also on the quality and accident of the voice. The boundary to the duration of syllable sound in discourse is not necessarily the limit to a stammerer's utterance, as is generally believed. The laws which regulate syllabication are universal in their operation; they limit the syllables of the most civilized language as well as the barbaric; they are one thing. The laws which limit the stammerer's utterance take in more data to arrive at a conclusion, and are varied in each case; they are another thing. But the laws of syllabication will throw a light on defective utterance. I have not room, nor is it within my present object, to enter upon the demonstration of these laws. As part of the Philosophy of Speech it is an interesting study.

The quackery and mysticism in the cure of impediments of speech arises from the general ignorance of the philosophy of the human voice and speech. Explanations of their phenomena have been demanded, to administer to the necessities of defective utterance, and to satisfy the ear of taste in a just elocution, but in vain. Bread has been asked for, and the stone has been given. The subject called for examination by accurate observation and experiment, not for the transcription of Greek and Roman knowledge, as if it were impossible for us to do more than quote classic authority. The fact appears scarcely credible, that when knowledge is within reach—when it can be obtained from its very source—from its fountain, with the trouble only of precise perception, we have hitherto been content to take it from its streamlets in its vague generalities, and that mudded and contaminated by the prejudice and musical ignorance with which the ancients have disguised the little knowledge they appear to have possessed on this subject." 15.

And further on our author remarks:—

"Speech is produced by a series of voluntary muscular actions. When obstructed by the involuntary action of some organ, or misactions of others, it resolves itself into a want of dexterity in the use of those muscles employed in speech; and their use can only be acquired by unremitting exercise, under the guidance of those who understand the circumstances under which they act. This is a work of time, and therefore not likely to be adopted, until every means promising a cure in a few days or weeks shall have been tried, and found unsuccessful. And when as much time has been wasted in observing the empirical oath-bound-secret directions as would probably have effected a cure, he is so disgusted then with even the idea of further trouble, that he is scarcely willing to commence a course of progressive exercises—to begin *de novo* to learn on principle his own oral language; to acquire it through the drudgery of a strict discipline in a course of vocal and enunciative gymnastics." 16.

Mr. Cull observes, and with some shew of reason, that in any analogous instance, say the fingering of a musical instrument, the individual is taught to acquire the necessary degree of art by proper tuition and general practice. He is not told to hold his finger in one position, or to cease playing for a certain time. No one in his senses would dream of teaching a pupil to play upon an instrument thus. Yet persons who stammer, are supposed to be likely to attain a due command over the vocal muscles and organ by empirical directions of this nature.

Mr. Cull introduces his plan by a reference to two cases of stammering; one of which was cured by elocutionary practice and instruction. The first case was that of a gentleman known to the late Dr. Mason Good. This gentleman was one of the worst stutterers Dr. Good had known, yet he was at the same time one of the best readers of Milton's Paradise Lost.

The second case was that of a gentleman known to our author and to Dr. Birkbeck. He could not read prose, nor converse without stammering, yet the late Mr. Thelwall, a professor of elocution, effected a perfect cure.

The following brief quotation may, perhaps, serve to introduce Mr. Cull's mode of procedure.

"The fact that some with impediments of speech can sing, read poetry, and declamatory composition, without interruption, is well known; it is of common occurrence. But if such cannot read or even sing, while a friend is attentive to their attempts, they can accomplish either, if a noise exist during their efforts. If a continued note be produced, as in drawing the bow across a violoncello-string, the power of utterance is augmented. If a piece of slow music of well-defined rhythm be played, the facility is increased. If a person read the same composition simultaneously with them in a rich sonorous voice, the facility is again heightened, which is rendered still more facile by simultaneously reading a third or fifth in the gamut below their tones; but while they are thus glibly reading, if the accompaniment cease, the impediment as instantly recurs." 19.

We all see through our own glasses. Mr. Cull looks at the story of Demosthenes, spouting at the sea with his mouth filled with pebbles, through his own peculiar spectacles. He thinks it probable that defective rhythm was the cause of Demosthenes' hesitation, and that, like the case quoted, and many others similar, cured by Mr. Thelwall by the correction of their rhythm, this in the case of Demosthenes, might be effected by his unconscious imitation of the rhythmical sound of the waves, dashing at periodic intervals upon the rocky shore. However this may be, the correction of the stammerer's "rhythm" is a paramount object with Mr. Cull. Elocutionary exercises, then, are brought into requisition by our author to educate the voice, and give it that force and direction which constitute the rhythm in question. His position, in short, is, that the acquisition of dexterity of the muscles employed in speech, through the diligent practice of vocal and enunciative exercises, is the only certain mode of permanently removing impediments of speech.

He analyses the manner in which an impediment occurs. He supposes the individual to have hesitated once; the sound on which he hesitated presents itself again, and again he stammers, from three causes :—

1. The memory of the hesitation;
2. The doubt of its present utterance; and
3. The hesitation.

Now, if we can introduce a new term, a knowledge of the means to utter a sound or combination of sounds which hitherto have impeded or stopped the current of utterance, the sequence will be changed, and if diligence only be used in submitting our new term to the influence of the laws of suggestion, to be confirmed into habit, the impediment never need again appear; because we have now a tripartite remedy for the previous tripartite hesitation.

We have—

1. The memory of the hesitation;
2. The knowledge of the means of utterance; and
3. Its application.

Of course habit must confirm the lesson taught and learned. In all cases, says our author, the application of elocutionary principles to obtain steadiness of voice, and regularity of action of the vocal organ, have alone mitigated the impediment, and when the vocal qualities have been used to simple elements, whether produced by appulsion or divulsion of the organs concerned in their mechanism, the effect has uniformly been the acquisition of power to enunciate every element without hesitation, and proceeding onwards, the varied articulations of these elements in every possible combination, forming speech at the bidding of the will. In this way the difficulty is not merely evaded, it is actually removed; and so far from there remaining any peculiarity of drawling, or mannerism, there is a sharpness in the speech, and command over the whole organs, so as to produce at will a vocal delineation of the several thoughts and their relations constituting the sense of an author. Nay, Mr. C. assures us that not only young persons, but adults also, have been cured by the means he recommends and adopts. His "vocal gymnasium" has been of great service to the ancient stammerer.

We have now done all we are able to do, in explaining the general principles of Mr. Cull. That they *are* general principles is evident. We do not suppose that their application will prove successful in all cases—perhaps they may not be permanently successful in the majority. But, founded in reason and in the spirit of philosophy, they cannot be injurious, and, in many cases, may be competent to release an individual from what must be regarded a very serious calamity.

It may be asked if this is all—if Mr. Cull does not offer some particular rules. He does not. At the hands of disreputable persons, his reasons would be unsatisfactory—his practice tainted with the suspicion of empiricism. But in this instance the excuse is judicious, and no doubt sincere—it is founded on the impossibility of the conveying complicated instructions in elocution, in terms that would at once be clear and comprehensive. We recommend the present pamphlet to those who stammer, and to those who are interested, and what physician is not, in understanding the causes, and being acquainted with the principles, that ought to regulate the treatment of impediments of speech.

